# Symptom Severity, Self-efficacy and Treatment-Seeking for Mental Health Among US Iraq/Afghanistan Military Veterans

**DOI:** 10.1007/s10597-020-00578-8

**Published:** 2020-02-17

**Authors:** Mary Keeling, Nicholas Barr, Hazel Atuel, Carl A. Castro

**Affiliations:** 1 Department of Health and Social Sciences, Faculty of Health and Applied Sciences, University of the West of England, Frenchey Campus, Coldharbour Road, Bristol, BS16 1QY UK; 2 Center for Innovation and Research on Veterans & Military Families, USC Suzanne Dworak-Peck School of Social Work, University of Southern California, 1150 South Olive Street, Suite 1400, Los Angeles, CA 90015 USA

**Keywords:** Treatment-seeking, Health belief model, Symptom severity, Self-efficacy, Military veterans

## Abstract

Military veterans have high rates of mental health problems, yet the majority do not seek treatment. Understanding treatment-seeking in this population is important. This study investigated if symptom severity and self-efficacy are associated with treatment-seeking among US Iraq/Afghanistan veterans. Survey data from 525 veterans meeting clinical criteria for PTSD and depression were included of which, 54.4% had sought treatment in the past 12 months. Multivariate logistic regression analysis indicated that high symptom severity was associated with treatment seeking, whereas high self-efficacy was associated with a decreased likelihood to seek treatment. Self-efficacy could be an underlying mechanism of treatment seeking decisions.

Evidence indicates that military personnel and veterans experience mental health conditions at elevated rates (Hoge et al. 2004; Seal et al. 2009; Thomas et al. 2010). For example, Seal et al. (2009), found in their study with 289,328 Iraq and Afghanistan veterans entering Veterans Affairs (VA) health care from 2002 to 2008, that mental health diagnoses increased substantially after the start of the Iraq war, especially among veterans younger than 25 years old. Yet, consistent with the general population, the prevalence of treatment-seeking for mental health problems is between 23 and 58 percent (Clement et al. 2014; Henderson et al. 2013; Ouimette et al. 2011; Porcari et al. 2017; Rosen et al. 2011; Stecker et al. 2007). Considering that, for the most part, both military personnel and veterans have access to a universal healthcare system, understanding what might increase or decrease the propensity to seek treatment for mental health problems among military veterans is an important area of investigation.

The Health Belief Model (HBM) (Rosenstock et al. 1988) attempts to explain health behaviors such as treatment-seeking and states that health related action depends on the simultaneous occurrence of three factors: 1. Sufficient health concern to make the health concern salient or relevant, such as experiencing severe symptoms. 2. Threat perception, which is the belief that one is susceptible or vulnerable to a serious health problem, such as impaired functioning. 3. The belief that engaging in an adaptive health behavior, such as seeking treatment, would be beneficial in reducing the threat, minus perceived costs and barriers, such as the cost/barrier of experiencing stigma.

An additional factor of the HBM is self-efficacy. Self-efficacy is an individual’s belief in his or her capacity to execute behaviors necessary to produce specific performance attainments and reflects an individual’s confidence in their ability to exert control over their motivation, behavior, and social environment (Bandura 1982). Rosenstock et al. (1988) proposed that to understand health behavior related to conditions requiring long term changes, such as accessing mental health care, consideration of the confidence individuals have in their ability to engage in such health behavior plays a role. As well as the simultaneous occurrence of the three factors described, individuals must also feel competent and confident (self-efficacious) to implement that change (Rosenstock et al. 1988).

Evidence for the first two factors of HBM are established in the literature investigating treatment-seeking for mental health among veterans. Symptom severity and greater impairment are consistently reported to be associated with seeking treatment for mental health problems among OIF/OEF veterans (DeViva et al. 2016; Hines et al. 2014; Porcari et al. 2017; Rosen et al. 2011). Evidence for the cost/benefit factor related to stigma and treatment-seeking described by the HBM has however, received inconsistent support, possibly due to methodological challenges (Sharp et al. 2015). Evidence for the role of self-efficacy in seeking treatment among military and veteran populations has not been examined.

Evidence for the role of self-efficacy in treatment-seeking from other populations is limited and inconsistent. Inverse to the predictions made by Rosenbeck et al. (1988), Jackson et al. (2007) found that lower self-efficacy was associated with a higher likelihood to seek treatment among a rural general population sample. However, a Swedish study found no association between general self-efficacy and treatment-seeking, but did find that lower self-efficacy was associated with being more likely to report barriers to care including, “it will pass by itself” and “I did not know where to get treatment” (Andersson et al. 2014).

Based on the limited investigation of the role of self-efficacy in veteran populations, the inconsistent results from other populations and, the proposed important role self-efficacy may play in decisions to seek treatment, investigation of self-efficacy and treatment seeking could prove fruitful for improving understanding of veterans’ treatment-seeking decisions. Moreover, self-efficacy presents a possible target of interventions designed to improve treatment seeking since it is a cognitive factor which is amenable to change (Andersson et al. 2014; Rosenstock et al. 1988). The current study therefore investigated the role of self-efficacy in decisions to seek treatment among a US veteran sample. Based on the HBM, this study will test two hypotheses:

## H1

Higher symptom severity will be associated with an increased likelihood to seek treatment.

## H2

Higher self-efficacy will be associated with an increased likelihood to seek treatment.

Whether the interaction between self-efficacy and symptom severity increases the likelihood to seek treatment will also be explored.

Finding a main effect of symptom severity on treatment-seeking will provide additional support for existing evidence and for an important factor of the HBM. If a main effect of self-efficacy on treatment-seeking is found this will support Rosenbeck et al.’s (1988) proposal to include self-efficacy in the HBM. Moreover, it would show that self-efficacy is a cognitive mechanism important in the treatment-seeking decisions of veterans. Investigating both hypotheses will help inform interventions aimed at increasing the propensity to seek treatment for mental health issues among military veterans.

## Method

### Sample and Recruitment

Veterans who had previously participated in veteran survey studies in Los Angeles County, Orange County, Chicago, and the San Francisco Bay area and had agreed to be re-contacted about future research participation opportunities were invited, via email, to participate in the current study (n = 2280). Eligibility criteria for participation included, having served since the terrorist attacks of September 11, 2001 (Post 9/11 veterans), and to have left military service (not currently be active duty).

### Data Collection Procedures

Invitations to participate were sent to the 2280 veterans via MailChimp. The email consisted of an invitation to participate, study information sheet, and a link to the online survey. The online survey was constructed in Qualtrics and all data was collected via this online platform. The beginning of the survey included eligibility questions which if not met the survey was terminated. A consent form followed, which had to be completed before the survey would continue. On completion of the survey, participants were redirected to an additional Qualtrics survey where they could provide their name and email address to receive a $15 gift card as compensation for their time. The contact information for the gift card was collected separately to ensure study data remained anonymous. Gift cards were emailed to those who completed the survey and provided their information. Their contact information was subsequently removed from the mailing list to ensure they were not contacted further to participate.

Participant recruitment lasted for 10 months, May 2016 to February 2017. The MailChimp invite was resent to all veterans who had not already provided information to receive the gift card, approximately twice a month. Some veterans contacted the research team to inform them that they were not eligible to take part. Their contact information was removed to avoid further contact.

### Measures

#### Demographics

Age, gender, marital status, employment status, education, type of military discharge, and eligibility to receive VA benefits and services.

#### Posttraumatic Stress Disorder

PTSD was measured using the Posttraumatic Stress Disorder Checklist for DSM-5 (PCL-5) (Weathers et al. 2013). The PCL-5 is a 20 item self-report measure assessing the 20 DSM-5 symptoms of PTSD. The PCL-5 demonstrates strong internal consistency (Cronbach’s α = 0.96), test–retest reliability (r = 0.84), and convergent and discriminant validity with a US veteran population (Bovin et al. 2016). Within this study sample strong internal consistency was also found (Cronbach’s α = 0.96). Symptom severity score is obtained by summing the scores for each of the 20 items (range 0–80). A PCL-5 cut point score of 33 is the clinical cutoff for this scale.

#### Depression

Depression was measured using the Patient Health Questionnaire 9 (Kroenke et al. 2001), a 9-item measure on a 4-point Likert type scale. Scores on the PHQ-9 range from 0 to 27 with a clinical cut point score of 10 or above. The PHQ-9 has strong internal consistency (Cronbach’s α = 0.89) and test–retest reliability (r = 0.84) (Kroenke et al. 2001). Within this study sample a similar internal consistency is found (Cronbach’s α = 0.88).

#### General Self-efficacy

Self-efficacy was measured using the General Self-Efficacy Scale which is a 10 item scale. Participants are asked to rate on a four-point Likert scale, ranging from “not at all true” to “exactly true”, how true ten statements are, for example “I can always manage to solve difficult problems if I try hard enough”. The scale was created to assess the general sense of perceived self-efficacy a person has of themselves. The scale has good internal consistency (Cronbach’s α = 0.86) (Schwarzer and Jerusalem 1995). Within this study’s sample good internal consistency was also found (Cronbach’s α = 0.91).

#### Treatment-Seeking

Treatment-seeking was measured by asking the question: “Have you sought treatment for a mental, emotional, or stressful problem in the last year from a mental health professional (e.g. Psychiatrist, Psychologist, Social Worker, VA Clinician?)” with response options “yes” or “no”.

### Statistical Analysis

Since this study is interested in investigating treatment-seeking for mental health problems, only those who scored above the clinical cutoff for either PTSD or depression were included in the analysis. The predictor variable symptom severity was measured as depression severity (total score on the PHQ-9) and PTSD severity (total score on the PCL-5). It is possible that severity of the different diagnoses effects treatment-seeking differently, therefore, the effect of depression symptom severity and PTSD symptom severity were investigated separately. Two samples were created; one including all participants who scored above the clinical cutoff on the PCL-5 only and one including all participants who scored above the clinical cutoff on the PHQ-9 only. The hypotheses were tested separately with each sample. Of note, 82 percent of the participants had co-morbid conditions and were included in both samples. All statistical analysis was conducted using SPSS Version 22.

To test both hypotheses, logistic regression analysis was conducted with treatment-seeking as the outcome variable. Initially each of the two predictors, symptom severity, measured as either PCL-5 score of PHQ-9 score, and general self-efficacy, were examined individually via univariate logistic regression analysis. Multivariate analysis logistic regression analysis with both predictors in the model was then conducted, followed by a multivariate analysis including demographic variables thought to be potentially associated with treatment-seeking based on a priori theory (age, gender, marital status). The covariates that were not significant in the model were removed after examining model fit. Interaction terms were included in the models as post hoc analyses to test for an interaction between symptom severity and self-efficacy.

### Ethics

The study received approval from the (masked for review) Institutional Review Board and was considered exempt since study datum would be anonymous. This study was therefore performed in accordance with the ethical standards laid down in the 1964 Declaration of Helsinki and its later amendments. All participants provided informed consent prior to inclusion in the study and all data is anonymous.

## Results

The survey was completed by 576 veterans, with a response rate of 25.3%. Whilst this response rate is low, it is likely due to the nature of the study focusing on mental health and treatment-seeking, therefore those with mental health experiences may have been more motivated to participate, compared to those who do not, as reflected in the high prevalence of PTSD and depression in the sample.

Of the 576 participants, 525 met the cut off for being a probable case for either PTSD and/or Depression. Only these 525 were included in the study. Demographic characteristics of the sample are shown in Table 1. Consistent with the profile of military veterans the majority were male, white, and educated to Bachelor’s level. Only 5.5% were not eligible to use VA services. As there was only one transgender veteran in the sample, and a priori theory indicates that gender is associated with treatment seeking, this participant was excluded from further analysis. Due to small numbers in the not VA eligible category, this variable was not included in further analysis. Of the 525 participants, 524 (99.8 percent) responded to the question asking if they had sought treatment for a mental, emotional, or stressful problem in the last year from a mental health professional, with 54.4 percent reporting they had sought treatment.

**Table 1:** Sample characteristics

Demographic	Total % (n)	Distribution of treatment seeking % (n)	
		No	Yes
Total		45.6 (239)	54.4 (285)
Age			
Mean (SD)	33.9 (8.62)	37.7	39.9
Range	21–73		
Gender			
Male	79.4 (417)	48.1 (200)	51.9 (216)
Female	20.4 (107)	36.4 (39)	63.6 (68)
Transgender	0.2 (1)	0.0 (0)	100.0 (1)
Ethnicity			
White	48.6 (255)	38.2 (97)	61.8 (157)
Black or African American	10.7 (56)	60.7 (34)	39.3 (22)
Hispanic/Latino	26.1 (137)	52.6 (72)	47.4 (65)
Other	14.7 (77)	46.8 (269)	54.4 (285)
Education			
High school diploma or less	12.6 (66)	15.2 (10)	84.8 (56)
Some college	20.8 (109)	53.7 (58)	46.3 (50)
Associate’s degree	17.3 (91)	60.4 (55)	39.6 (36)
Bachelor’s degree or higher	49.3 (259)	44.8 (116)	55.2 (143)
Marital status			
Single	16.8 (88)	44.3 (39)	55.7 (49)
Married, domestic partner, long term	65.9 (346)	49.0 (169)	51.0 (176)
Divorced, separated, windowed	17.1 (90)	34.4 (31)	65.6 (59)
VA services eligible			
No	5.5 (29)	93.1 (27)	6.9 (2)
Yes	87.6 (460)	41.6 (191)	58.4 (268)
Above PCL-5 cutoff			
No	11.2 (57)	82.5 (47)	17.5 (10)
Yes	88.8 (450)	40.3 (181)	59.7 (268)
Above PHQ-9 cutoff			
No	1.0 (5)	60.0 (3)	40.0 (2)
Yes	99.0% (509)	45.9 (233)	54.1 (275)

*NB* numbers may not add up due to missing data

### Univariate Analysis of Symptom Severity and Treatment-Seeking

The mean PCL-5 score among the veterans in the PTSD sample was 63.9 (16.2 SD; 33–100 range). Using the PTSD sample, in a model with 448 cases analyzed, univariate logistic regression analysis indicated that PTSD symptom severity was positively associated with treatment-seeking. The mean PHQ-9 score among the veterans in the depression sample was 21.1 (6.4 SD; 10–36 range). Using the depression sample, in a model with 507 cases analyzed, univariate logistic regression analysis also indicated that depression symptom severity was positively associated with treatment-seeking (Table 2).

**Table 2:** Coefficients and Odds Ratios with 95% confidence intervals (CI) of the model investigating the association of PTSD symptom severity and depression symptom severity on treatment-seeking

	B (S.E.)	Adjusted OR (95% CI)
PTSD symptom severity	0.031	1.03 (1.02–1.04)*
Constant	− 1.571	
Depression symptom severity	0.103	1.11 (1.08–1.14)*
Constant	− 1.999	

PTSD model—R^2^ = .056 (Cox and Snell) .075 (Nagelkerke). Model *X*^2^ (1) = 25.63; *p* ≤ .001. Depression model—R^2^ = .092 (Cox and Snell) .12 (Nagelkerke). Model *X*^2^ (1) =  48.92; *p* ≤ .001. *p < .001

### Univariate Analysis of Self-efficacy and Treatment-Seeking

In the sample of veterans reporting the clinical cut-off for PTSD (n = 450), 440 answered all of the questions on the general self-efficacy scale with a mean score of 27.7 (5.9 SD; 10–40 range). Using this PTSD sample, univariate logistic regression analysis including a model with 439 cases analyzed, indicated that general self-efficacy has a negative direct association with treatment-seeking.

In the sample of veterans reporting the clinical cut-off for depression (n = 509), 501 answered all of the questions on the general self-efficacy scale with a mean score of 28.4 (6.02 SD; 10–40 range). Using the sample of those above the clinical cutoff for depression, a univariate logistic regression analysis, including a model with 500 cases analyzed, indicated that general self-efficacy has a negative direct association with treatment-seeking (Table 3).

**Table 3:** Coefficients and Odds Ratios with 95% confidence intervals (CI) of the association between general self-efficacy and treatment-seeking in samples with those with probable PTSD and those with probable depression

	B (S.E.)	Adjusted OR (95% CI)
PTSD sample		
General self-efficacy	− .145	.87 (.83–.90)*
Constant	4.481	
Depression sample		
General self-efficacy	− .163	.85 (.82–.88)*
Constant	4.860	

PTSD model—R^2^ = .126 (Cox and Snell) .171 (Nagelkerke). Model *X*^2^ (1) = 23.93; *p* ≤ .01. Depression model—R^2^ = .163 (Cox and Snell) .218 (Nagelkerke). Model *X*^2^ (1) =  89.02; *p* ≤ .001. *p < .001

### Multivariate Analysis of the Effect of Symptom Severity and Self-efficacy on Treatment-Seeking

#### PTSD Sample

To examine hypotheses 1 and 2, multivariate logistic regression analysis was conducted using the PTSD sample. In a model with 439 cases analyzed, the multivariate analysis indicated that both PTSD symptom severity (1.03 OR; 1.01 – 1.04 95% CI) and general self-efficacy (0.87 OR; 0.83–0.91 95% CI) had a positive main effect on treatment-seeking. Age, gender, and marital status were added to the model as co-variates however, only gender was significant and included in the final analysis. Multivariate logistic regression analysis with 439 cases analyzed, indicated that PTSD symptom severity had a positive main effect on treatment-seeking and self-efficacy had a negative main effect on treatment-seeking, with gender as a covariate indicating that women are more likely to seek treatment (Table 4). These results provide evidence of the acceptance of hypotheses one for those with probable PTSD. However, hypotheses two is only partially supported, as although a direct main effect was found, it was in the direction opposite to predicted.

**Table 4:** Coefficients and Odds Ratios with 95% CI of a multivariate logistic regression analysis investigating the main effect of PTSD symptom severity and general self-efficacy on treatment-seeking with the inclusion of age and gender as covariates

	B (S.E.)	Adjusted OR (95% CI)
PTSD symptom severity	.025	1.03 (1.01–1.04)**
General self-efficacy	− .135	.87 (.84–.91)**
Gender		
Male		
Female	.734	2.08 (1.16–3.73)*
Constant	2.527	
Depression symptom severity	.068	1.07 (1.04–1.11)**
General self-efficacy	− .148	.86 (.83–.90)**
Age	.028	1.03 (1.00–1.05)*
Marital status		
Single (base)	–	–
Married, domestic partner, long term	− .680	.51 (.29–.89)*
Divorced, separated, windowed	− .249	.78 (.377–1.62)
Constant	2.450	

PTSD model—R^2^ = .167 (Cox and Snell) .226 (Nagelkerke). Model *X*^2^ (1) = 80.34; *p* ≤ .01. Depression model—R^2^ = .208 (Cox and Snell) .278 (Nagelkerke). Model *X*^2^ (1) = 116.25; *p* ≤ .001. *p < 0.05; **p < 0.001

#### Depression Sample

Hypotheses 1 and 2 were also tested using the depression sample. In a model with 482 cases analyzed, multivariate logistic regression analysis indicated that both depression symptom severity (1.07 OR: 1.04–1.11 95% CI) and general self-efficacy (0.87 OR: 0.83–0.90 95% CI) had a positive main effect on treatment-seeking. Age, gender, and marital status were added to the model as covariates and indicated that age and marital status made a significant contribution to the model. Multivariate logistic regression analysis with 499 cases analyzed, indicated that depression symptom severity had a positive main effect on treatment-seeking and self-efficacy had a negative main effect on treatment-seeking with the covariates indicating that those who were older were more likely to seek treatment, and those who were married were less likely to seek treatment compared to those who were single (Table 4). These results support H1. However, H2 was partially supported because even though a direct main effect was found, it was in the direction opposite to what was predicted.

### Is There an Interaction Effect Between Symptom Severity and General Self-efficacy?

An interaction term was added to the multivariate logistic regression models (excluding the covariates) for both the probable PTSD and depression samples. Results of these analyses indicated that the interaction effects in both samples were not significant (Table 5).

**Table 5:** Coefficients and Odds Ratios with 95% CI of a multivariate logistic regression analysis investigating the interaction effect of PTSD symptom severity and general self-efficacy on treatment-seeking

	B (S.E.)	Adjusted OR (95% CI)
PTSD symptom severity	– .036	.96 (.89–1.04)
General self-efficacy	– .281	.76 (.63–.91)*
PTSD*GSE	.002	1.00 (1.00–1.01)^††^
Constant		
Depression symptom severity	– .105	.90 (.75–1.09)
General self-efficacy	– .273	.76 (.66–88)**
Depression*GSE	.006	1.00 (1.00–1.01)^†^
Constant	6.849	

PTSD model—R^2^ = .160 (Cox and Snell) .216 (Nagelkerke). Model *X*^2^ (1) = 76.39; *p* ≤ .01. Depression model—R^2^ = .197 (Cox and Snell) .264 (Nagelkerke). Model *X*^2^ (1) = 109.90; *p* ≤ .001. *p < 0.05; **p < 0.001; ^†^068; ^††^.112

## Discussion

Guided by the Health Belief Model (HBM) (Rosenstock et al. 1988), this study examined the associations of symptom severity and self-efficacy on treatment-seeking among a sample of post 9/11 veterans. The overall prevalence of treatment-seeking (54.4%) among this study sample (n = 525) is consistent with the prevalence of treatment-seeking found in other studies with similar veteran samples (DeViva et al. 2016; Porcari et al. 2017; Rosen et al. 2011).

Hypothesis 1 tested whether symptom severity had a positive main effect on treatment-seeking and was confirmed for PTSD symptom severity and depression symptom severity. Consistent with previous literature and the HBM (Adler et al. 2015; DeViva et al. 2016; Porcari et al. 2017; Rosen et al. 2011), the results from this study indicate that higher depression and PTSD symptoms are associated with an increased likelihood that veterans will seek treatment.

Hypotheses 2 tested whether general self-efficacy was associated with treatment-seeking as per the suggestion of Rosenstock et al. (1988) in terms of the HBM. In both the depression and PTSD samples a main effect was found but in the opposite direction predicted. Rather than increased self-efficacy being associated with an increased likelihood of treatment-seeking, increased self-efficacy was associated with a decreased likelihood of treatment-seeking. This is consistent with the results of a study conducted with a rural general population sample that found that lower self-efficacy was associated with an increased likelihood to seek treatment (Jackson et al. 2007). This association between self-efficacy and treatment-seeking requires further investigation and replication with other veteran and non-veteran populations.

One of the most frequently reported barriers to care found in existing research with veterans (Adler et al. 2015; Keeling et al. 2017; Momen et al. 2012; Sayer et al. 2009; Stecker et al. 2007), and in some civilian studies (Mojtabai et al. 2011), is having a preference for managing their problems on their own. Arguably, self-management requires high self-efficacy and confidence in one’s ability to manage the problem independently. Therefore, evidence that high self-efficacy is associated with a decreased likelihood to seek treatment may be due to individuals’ confidence in their ability to take care of the problem themselves and a perceived lack of need for external treatment. A preference for managing one’s problems on their own, is often considered to be related to the military culture of “soldiering on” and the military ethos of self-reliance (Murphy et al. 2014). It is possible that this ethos creates a double edge sword, that while this might be good during military service, it may impact military personnel and veterans’ propensity to seek treatment, instead feeling equipped and preferring to manage the problem independently without professional treatment. The association between self-efficacy, a preference for self-management and treatment-seeking requires further investigation.

## Limitations

This study is cross-sectional therefore causation and direction of effect is not guaranteed and the results should be interpreted with this consideration. Self-efficacy was measured using a general measure, rather than a measure specific to self-efficacy of seeking treatment for mental health concerns. Bandura (1982) recommends using measures of self-efficacy specific to the behavior or action under investigation. The lack of use of a treatment-seeking specific measure could have impacted our results. However, a robust measure of treatment-seeking self-efficacy was not available at the time the study was conducted. The odds ratios for the association between PTSD symptoms and depression symptoms, despite being highly significant, are close to 1.00 therefore this result should be interpreted with this in consideration since the strength of association is small.

Otherwise, this study used well validated and robust measures with a large sample of US veterans. The sample demographics are consistent with the veteran population and the prevalence of treatment-seeking was consistent with existing literature, indicating our sample is likely to be representative of the urban veteran population. Studies investigating veterans in rural areas may find different results since rural areas present additional barriers to care. A final strength of this study was that unlike many other studies it did not recruit veterans via VA health services.

### Implications and Recommendations

The results from this research indicate two distinct factors associated with treatment-seeking among US post 9/11 veterans and should be considered when considering ways to improve treatment-seeking for mental health concerns among veterans. Firstly, efforts and interventions directed at improving veterans’ ability to recognize their symptoms as treatable and manageable mental health conditions could lead to veterans seeking treatment when symptoms are less severe. As Seal et al. (2009) recommend, early interventions may prevent chronic mental illness.

Secondly, the finding that high self-efficacy is associated with a decreased likelihood to seek treatment, not only draws into question Rosenstock et al.’s (1988) inclusion of self-efficacy in the HBM, but suggests a possible cognitive mechanism important in decisions to seek treatment. A possible explanation for why high self-efficacy likely deters veterans from seeking treatment could be due to the commonly reported barrier of having a preference for managing the problem on their own. The association between self-efficacy and treatment-seeking requires replication in other studies with veteran populations and a possible reconsideration of the inclusion of self-efficacy in the HBM. Moreover, the association between self-efficacy, a preference for self-management, and treatment-seeking requires investigation.

Taken together, these have implications for the overall VA population and access to care. Educational resources made available to the VA population providing psychoeducation about the symptoms of and available treatments for PTSD symptoms and depression symptoms could help increase the propensity to seek treatment. Psychoeducation resources could help increase mental health literacy among the VA population, raise awareness of the experience of symptoms as treatable, and highlight the available support and treatments.

## Conclusion

This study examined the impact of symptom severity and self-efficacy on treatment-seeking for mental health among a US veteran sample. Consistent with previous research and the HBM, symptom severity appears to be a key component influencing treatment-seeking. Although programs designed to alleviate mental health stigma have been provided to military populations, programs targeting symptom identification may be an area of targeted intervention. It appears that self-efficacy is associated with treatment-seeking. Since evidence of this association is limited in the literature, further studies are required to replicate this finding. Moreover, studies to investigate the association between self-efficacy and specific barriers to care are also required. The results of this study provide initial evidence that self-efficacy could be an underlying cognitive mechanism important in decisions to seek- treatment.
